# Clinical evidence of acupuncture and moxibustion for irritable bowel syndrome: A systematic review and meta-analysis of randomized controlled trials

**DOI:** 10.3389/fpubh.2022.1022145

**Published:** 2022-11-24

**Authors:** Yuanming Yang, Kehan Rao, Kai Zhan, Min Shen, Huan Zheng, Shumin Qin, Haomeng Wu, Zhaoxiang Bian, Shaogang Huang

**Affiliations:** ^1^Dongguan Hospital of Guangzhou University of Chinese Medicine, Dongguan, China; ^2^The Second Clinical College of Guangzhou University of Chinese Medicine, Guangzhou, China; ^3^School of Acupuncture-Moxibustion and Tuina of Shandong University of Traditional Chinese Medicine, Jinan, China; ^4^The Second Affiliated Hospital of Guangzhou University of Chinese Medicine, Guangdong Provincial Hospital of Chinese Medicine, Guangzhou, China; ^5^Hong Kong Chinese Medicine Clinical Study Centre, School of Chinese Medicine, Hong Kong Baptist University, Hong Kong, Hong Kong SAR, China

**Keywords:** irritable bowel syndrome, acupuncture, moxibustion, complementary and alternative medicine, abdominal pain, quality of life

## Abstract

**Background:**

Acupuncture and moxibustion have been widely used in the treatment of Irritable Bowel Syndrome (IBS). But the evidence that acupuncture and moxibustion for IBS reduction of symptom severity and abdominal pain, and improvement of quality of life is scarce.

**Methods:**

PubMed, Embase, Cochrane Library, Web of Science, Chinese National Knowledge Infrastructure (CNKI), Chinese Scientific Journals Database (VIP), Wanfang Database, China Biomedical Literature Service System (SinoMed), and unpublished sources were searched from inception until June 30, 2022. The quality of RCTs was assessed with the Cochrane Collaboration risk of bias tool. The strength of the evidence was evaluated with the Grading of Recommendations Assessment, Development and Evaluation system (GRADE). Trial sequential analysis (TSA) was conducted to determine whether the participants in the included trials had reached optimal information size and whether the cumulative data was adequately powered to evaluate outcomes.

**Results:**

A total of 31 RCTs were included. Acupuncture helped reduce the severity of symptoms more than pharmaceutical drugs (MD, −35.45; 95% CI, −48.21 to −22.68; *I*^2^ = 71%). TSA showed the cumulative Z score crossed O'Brien-Fleming alpha-spending significance boundaries. Acupuncture wasn't associated with symptom severity reduction (SMD, 0.03, 95% CI, −0.25 to 0.31, *I*^2^ = 46%), but exhibited therapeutic benefits on abdominal pain (SMD, −0.24; 95% CI, −0.48 to −0.01; *I*^2^ = 8%) compared to sham acupuncture. Moxibustion show therapeutic benefits compared to sham moxibustion on symptom severity (SMD, −3.46, 95% CI, −5.66 to −1.27, *I*^2^ = 95%) and abdominal pain (SMD, −2.74, 95% CI, −4.81 to −0.67, *I*^2^ = 96%). Acupuncture (SMD, −0.46; 95% CI, −0.68 to −0.24; *I*^2^ = 47%) and the combination of acupuncture and moxibustion (SMD, −2.00; 95% CI, −3.04 to −0.96; *I*^2^ = 90%) showed more benefit for abdominal pain compared to pharmacological medications as well as shams. Acupuncture (MD, 4.56; 95% CI, 1.46–7.67; *I*^2^ = 79%) and moxibustion (MD, 6.97; 95% CI, 5.78–8.16; *I*^2^ = 21%) were more likely to improve quality of life than pharmaceutical drugs.

**Conclusion:**

Acupuncture and/or moxibustion are beneficial for symptom severity, abdominal pain and quality of life in IBS. However, in sham control trials, acupuncture hasn't exhibited robust and stable evidence, and moxibustion's results show great heterogeneity. Hence, more rigorous sham control trials of acupuncture or moxibustion are necessary.

**Systematic review registration:**

https://www.crd.york.ac.uk/PROSPERO/display_record.php?RecordID=262118, identifier CRD42021262118.

## Key points

- Numerous IBS patients seek substitute medical help from acupuncture and moxibustion. It is essential for physicians to understand the evidence supporting acupuncture and moxibustion as a treatment for IBS.
- In this systematic review and meta-analysis, acupuncture and/or moxibustion are beneficial for symptom severity, abdominal pain and quality of life. The strength of the evidence ranges from very low to high.
- Acupuncture hasn't exhibited robust and stable evidence, and moxibustion's results show great heterogeneity. However, high quality of sham-controlled trials are necessary to consolidate these results.

## Introduction

Irritable bowel syndrome (IBS) is the most common functional bowel disorder, which characterized by abdominal pain or discomfort association with altered stool form or frequency (1, 2). The disorder affects 5–17.5% of general population and has significant effect on quality of life and social function (2–4). Moreover, The pain and discomfort of abdominal and other impairment from IBS contribute to significant healthcare resource consumption and workplace absenteeism (5–7). The biopsychosocial mechanisms that explain abdominal pain and disordered bowel habits in IBS are multifaceted, including genetic predisposition, adverse childhood events, psychological factors, and changes in the enteric nervous system, which regulates intestinal motor, sensory, mucosal barrier, and secretory responses (1). Due to the complexity of the underlying mechanism, effective and safe medications are still in research. Most of the drugs recommended for treating the condition only focus on symptom alleviation, and antispasmodics, tricyclic antidepressants and selective serotonin reuptake inhibitors were found to have low to moderate quality of evidence by the American College of Gastroenterology Task Force. They also found some of these agents have a risk of ischemic colitis and cardiovascular events (8).

Patients and healthy providers are frequently dissatisfied with the existing pharmacological drugs and may seek complementary and alternative medicine (CAM) for help (9). Acupuncture and moxibustion, also termed energy-healing therapies, are two of the most widely utilized CAM therapies worldwide. Several systematic reviews and meta-analyses of acupuncture and moxibustion have been performed, however, the primary outcomes of these reviews were based on adequate relief rate or total response. IBS symptom severity score and abdominal pain, which are recommended by the FDA to assess the therapeutic effect (10), were rarely assessed as the primary or secondary outcomes in this meta-analysis. In addition, some conflicting results from previous meta-analysis still couldn't provide a definitive conclusion, for example, relative to sham acupuncture, real acupuncture had no significant benefit for symptom severity, but patients receiving real acupuncture reported greater improvements of IBS symptoms compared with patients receiving pharmacological therapies (11). It has been several years since the publication of the most recent meta-analysis for acupuncture and moxibustion in the treatment of IBS. More rigorous randomized controlled trials (RCTs) of acupuncture and related therapies have been published in recent years, for instance, a multicenter RCT of acupuncture published in 2021 found that patients with IBS who received acupuncture therapy experienced a significant reduction in IBS symptom severity and an improvement in quality of life (12). What's not mentioned in the previous meta-analysis is the sample size estimation, which is as important as sample size calculation in RCTs. And none of the previous meta-analysis had corrected the increased risk of type I errors caused by parse data and repeated significance testing on accumulating data.

In light of the conflicting results, limitations of previous reviews, the increasing number of RCTs of acupuncture or moxibustion used in IBS, and the ensuring need for critical evacuation, a systematic review and meta-analysis of the available evidence is essential. The specific research questions were as follows: do acupuncture or moxibustion contribute to reducing symptom severity and abdominal pain, and improving the quality of life compared with pharmacological medications or sham control?

## Methods

### Protocol and guidance

This systematic review and meta-analysis followed Preferred Reporting Items for Systematic Reviews and Meta-Analysis (PRISMA), and the protocol was registered in PROSPERO with the ID of CRD42021262118.

### Inclusion criteria

We considered trials to be eligible if they enrolled adults (age ≥18) with IBS; if they compared acupuncture or moxibustion with sham control or pharmacological medications (when other therapies were also given, they had to be the same dosage in all groups); if they provided information in symptom severity measured by the IBS symptom severity scale (IBS-SSS), abdominal pain, or IBS quality of life (IBS-QOL); and if they were randomized controlled trials.

### Exclusion criteria

We excluded studies that were case reports, case series, or observational studies; if the participants were pregnant or lactating women; if the intervention included laser acupuncture, non-invasive electrostimulation (i.e., using electrodes on the skin rather than needles to stimulate acupuncture points), transcutaneous electrical nerve stimulation, and acupressure; and if studies compared two types of acupuncture techniques or acupuncture with other traditional treatments. All conference proceedings, guidelines, dissertations, commentaries, and letters were excluded.

### Outcomes

The primary outcome was symptom severity, measured by the Irritable Bowel Syndrome Symptom Severity Scale (IBS-SSS). Secondary outcomes were abdominal pain and quality of life as measured by Irritable Bowel Syndrome Quality of Life (IBS-QOL).

### Search strategy

One of the authors (YYM) conducted the search of several databases: PubMed, Embase, Web of Science, Cochrane Library, the Chinese National Knowledge Infrastructure Database (CNKI), Wanfang Database, China Science and Technology Journal Database (VIP) and China Biomedical Literature Service System (SinoMed) from database inception to June 30, 2022. ClinicalTrials.gov was also searched to identify ongoing or unpublished eligible trials. The search strategy consists of 3 components: clinical condition (Irritable Bowel Syndrome), intervention (acupuncture or moxibustion) and study design (randomized controlled trial). To maximize the search of relevant articles, existing systematic reviews were examined to identify additional studies. Language restrictions were not applied.

### Study selection

**Figure 1:**
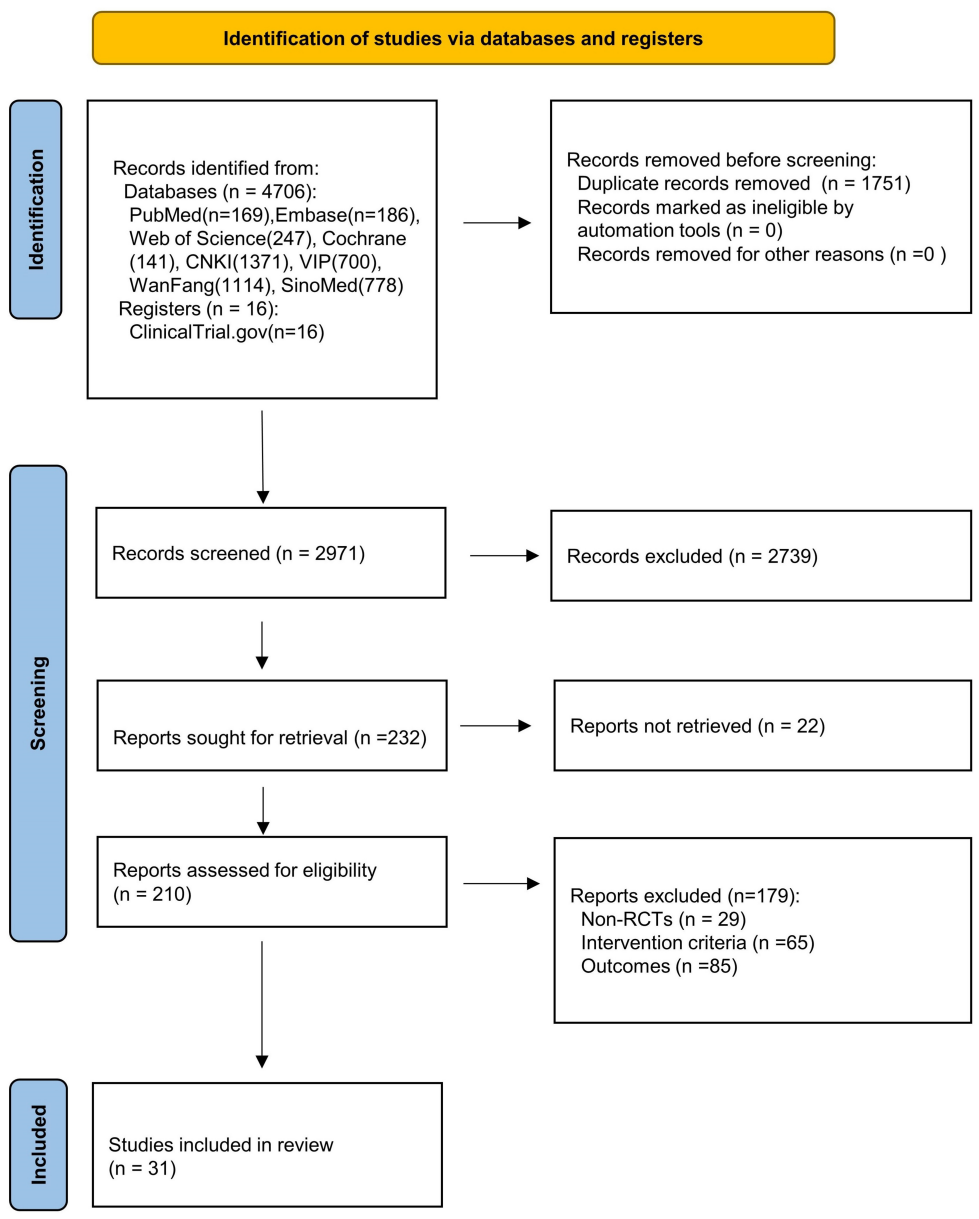
PRISMA 2020 flow diagram.

The searching results were exported to Endnote for screening and removing duplicates. Two reviewers (RKH and ZK) independently reviewed the titles and abstracts to identify any relevant studies. Citations deemed potentially relevant by either screener were advanced to second-stage full-text review. Then, full text reports were retrieved and screened for eligibility. Any discrepancies between the reviews were handled through discussion or consultation with a third party (SM) until consensus was reached. The flowchart illustrated the process of literature review and study selection (Figure 1).

### Data collection process

Two independent researchers (YYM and RKH) used a standard data extraction form to extract data from the included trials. We extracted the following information from included studies: study title, first author, demographic data, details of the interventions, and outcomes.

### Assessment of risk of bias and quality of evidence

Quality of all included trials was assessed independently by two investigators (YYM and SM) who recorded the method used to generate the randomization schedule and allocation concealment; whether blinding was implemented for participants, prescribers and outcome assessors; whether there was evidence of incomplete outcome data, and whether there were any selective outcome reporting data available. In addition, they examined the quality of evidence for outcomes using the Grading of Recommendations Assessment, Development and Evaluation (GRADE) approach.

### Data synthesis

RevMan (version 5.4.4; the Cochrane Collaboration) and Stata (version 16; Stata Corp, LLC) were used to perform statistical analyses. As the outcomes we selected were continuous variables, mean differences (MD) and 95% confidence intervals (CI) were calculated for variables with the same scale (e.g., symptom severity as measured by IBS-SSS and quality of life as measured by IBS-QOL). For continuous outcomes with different scales (e.g., different measures of abdominal pain), the standardized mean difference (SMD) and 95% confidence intervals (CI) were calculated. Heterogeneity was assessed using the *I*^2^ test. Fixed effects models were used to pool outcomes if significant heterogeneity was not present (*I*^2^ < 50%), otherwise, random effects models were applied. The possibility of small study effects was assessed by the Egger and Begg tests.

### Trial sequential analysis

Trial sequential analysis (TSA) was conducted to determine whether the cumulative data has significant power to evaluate outcomes. In meta-analysis, TSA can be used to assess the likely influence of future trials on the pooled findings and estimate the point at which further studies are not likely to change the pooled findings (13). In our research, TSA (version 0.9.5.10 Beta, Copenhagen Trial unit, https://ctu.dk/tsa/) was performed with an overall 5% risk of type I error and 80% power.

### Subgroup analyses and sensitively analyses

The studies were categorized by the type of intervention (acupuncture, moxibustion or the combination of acupuncture and moxibustion). Subgroup analyses were performed according to comparators (e.g., pharmacological medications including pinaverium, trimebutine, loperamide and others). Sensitive analyses were conducted by systematically omitting one of the trials.

## Results

### Eligible studies and study characteristics

A total of 4,706 records were identified through different database searches and 16 records were searched from ClinicalTrial.gov, from which 1,751 duplicate publications were removed and 2,940 articles were excluded for not meeting the inclusion criteria. Thirty-one eligible RCTs (12, 14–43) were included in the final meta-analysis, and details are shown in Table 1.

**Table 1:** Characteristics of randomized controlled trials.

**Study ID**	**Location**	**Number** **of** **patients**	**IBS details**		**Intervention**			**Control**			**Mean age (SD)**		**Gender** **(Male/Female)**	
			**Definition**	**Type**	**Method**	**Duration** **(weeks)**	**Follow-up** **(weeks)**	**Method**	**Duration** **(weeks)**	**Follow-up** **(weeks)**	**Interven-** **tion**	**Control**	**Interven-** **tion**	**Control**
Jing et al. (12)	China	137	Rome III	IBS-C	Acupuncture, 30 min, 3/weeks	6	18	Polyethylene glycol, 20 g, qd	6	18	46.34 (14.32)	47.02 (13.59)	33/59	17/28
Jing et al. (12)	China	382	Rome III	IBS-D	Acupuncture, 30 min, 3/weeks	6	18	Pinaverium, 50 mg, tid	6	18	45.72 (12.52)	46.99 (12.48)	146/106	71/59
Li et al. (27)	China	70	Rome III	IBS-D	Acupuncture, 20 min, 5/weeks	8	–	Trimebutine maleate, 0.2 g tid	8	–	38.15 (7.85)	38.45 (8.22)	12/23	13/22
Pei et al. (31)	China	519	Rome III	IBS-C/D	Acupuncture, 30 min, 3/weeks	6	18	Polyethylene glycol, 10 g, qd (IBS-C); Pinaverium, 50 mg, tid (IBS-D)	6	18	45.89 (13.01)	47.00 (12.73)	177/165	88/87
Wenjiao (36)	China	80	Rome III	IBS-D	Acupuncture, 30 min, 3/weeks	6	18	Pinaverium, 50 mg, tid	6	18	46.38 (11.47)	47.49 (12.39)	22/18	19/21
Guojuan (17)	China	70	Rome IV	IBS-D	Acupuncture, 30 min, 5/weeks	4	–	Pinaverium, 50 mg, tid	4	–	39.3 (11.5)	38.4 (13.5)	16/19	13/22
Lei (24)	China	104	Rome III	IBS-D	Acupuncture, 30 min, 5/weeks	4	–	Pinaverium, 50 mg, tid live combined Bifidobacterium, Lactobacillus and Enterococcus powder, 2 g, tid	4	–	40.71 (10.84)	41.55 (11.62)	24/30	23/27
Hongming et al. (20)	China	70	Rome III	IBS-D	Acupuncture, 30 min, 5/weeks	16	24	Pinaverium, 50 mg, tid Bifidobacterium capsules, 0.84 g, tid	16	24	44.38 (8.61)	44.05 (8.72)	16/19	17/18
Yu et al. (40)	China	58	Rome III	IBS-D	Acupuncture, 30 min, 3/weeks	4	12	Pinaverium, 50 mg, tid	4	12	41 (11)	39 (12)	14/16	11/17
Jing et al. (22)	China	77	Rome III	IBS-D	Acupuncture, 30 min, 3/weeks	6	–	Pinaverium, 50 mg, tid	6	–	46 (13)	48 (13)	24/27	13/13
Wenjing and Yingjie (37)	China	80	Rome III	IBS-D	Acupuncture	4	–	Pinaverium, 50 mg, tid	4	–	30.0 (6.2)	32.2 (5.4)	12/28	15/25
Lixia et al. (26)	China	60	Rome III	IBS-C	Acupuncture, 30 min, 5/weeks	4	12	lactulose oral liquid, 15 ml, tid	4	12	44 (12)	44 (14)	11/19	9/21
Hao et al. (18)	China	70	Rome III	IBS-D	Acupuncture, 30 min, 3–4/weeks	4	–	Pinaverium, 50 mg, tid	4	–	39.1 (11.8)	37.9 (11.5)	15/20	18/17
Sun et al. (34)	China	63	Rome III	IBS-D	Acupuncture	4	–	Pinaverium, 50 mg, tid	4	–	38.81 (11.80)	38.59 (11.45)	13/18	20/12
Bin et al. (15)	China	54	Rome II	IBS-D	Acupuncture, 30 min, 3/weeks	8	–	Pinaverium, 50 mg, tid	8	–	–	–	12/15	13/14
Mak et al. (29)	Hong Kong	80	Rome III	IBS-D	Acupuncture, 30 min, 1/weeks	10	16	Sham acupuncture, 30 min, 1/weeks	10	16	50.85 (11.57)	50.83 (14.15)	20/20	18/22
Lowe et al. (28)	Canada	79	Rome I	-	Acupuncture, 30 min, 2/weeks	4	12	Sham acupuncture, 30 min, 2/weeks	4	12	42 (15)	43 (15)	7/36	10/26
Xia et al. (38)	China	80	Rome III	IBS-D	Acupuncture, 40 min; Bifidobacterium tetralogy capsule, 4.5 g, tid	4	–	Sham acupuncture, 40 min; Bifidobac-terium tetralogy capsule, 4.5 g, tid	4	–	40.0 (13.2)	37.6 (12.6)	23/17	21/19
Park and Cha (30)	South Korea	42	Rome III	–	Acupuncture, 25 min, 2/week	4	–	Sham acupuncture, 25 min, 2/week	4	–	22.26 (3.23)	21.48 (2.73)	–	–
Lembo et al. (21)	USA	153	Rome II	Any	Acupuncture	3	–	Sham acupuncture	3	–	37.5 (14.6)	38.9 (14.1)	–	–
Wei et al. (35)	China	80	Rome IV	IBS-D	Moxibustion, every other day	4	–	Pinaverium, 50 mg, tid	4		43 (4)	43 (5)	16/24	18/22
Yanli (39)	China	52	NA	IBS-D	Moxibustion, 30 min, bid plus Loperamide 2 mg, bid	2	–	Loperamide 2 mg, bid	2	–	41.33 (1.14)	22/30 (M/F)	–	–
Lingjun et al. (25)	China	80	Rome III	IBS-D	Moxibustion, qd	4	–	Trimebutine maleate, 0.2 g tid	4	–	41.33 (1.14)	44.05 (1.14)	12/28	14/26
Di et al. (16)	China	97	Rome III	IBS-D	Moxibustion, qd	4	12	Loperamide 2 mg, tid	4	12	43.11 (13.22)	44.53 (12.63)	21/28	26/22
Shouqin (33)	China	60	Rome III	IBS-D	Moxibustion, 45 min Pinaverium, 50 mg, tid	4	8	Pinaverium, 50 mg, tid	4	8	35.68 (8.25)	26.23 (7.82)	13/17	11/19
Haoran et al. (19)	China	60	Rome II	IBS-D	Moxibustion, 30 min	2	–	Loperamide, 2 mg, bid	2	–	48.3 (12.5)	46.8 (13.2)	23/7	24/6
Jun (23)	China	100	Rome III	IBS-D	Acupuncture and moxibustion, 30 min	4	–	Pinaverium, 50 mg, tid	4	–	44.8 (9.5)	45.3 (10.2)	21/29	20/30
Qian et al. (32)	China	111	Rome IV	IBS-C/D	Acupuncture and moxibustion, 20 min,	4	–	Trimebutine maleate, 0.2 g tid	4	–	47.00 (2.50)	46.80 (2.70)	25/31	23/32
Anastasi et al. (14)	USA	29	Rome II	NA	Acupuncture and moxibustion	4	–	Sham acupuncture and moxibustion	4	–	47.1	34.3	6/9	5/10
Shen et al. (43)	China	65	Rome III	IBS-D	Acupuncture, 3 times per week, 30 min	8	–	Sham acupuncture, 3 times per week, 30 min	8	–	38.61 (11.57)	43.28 (13.64)	18/15	16/16
Bao et al. (42)	China	104	Rome III	IBS-D	Moxibustion, 3 times per week, 30 min	6	24	Sham moxibustion, 3 times per week, 30 min	6	24	47.6 (11.9)	45.2 (14.7)	23/29	28/24
Wang et al. (41)	China	76	Rome IV	IBS-D	Moxibustion, 3 times per week, 30 min	6	12	Sham moxibustion, 3 times per week, 30 min	6	12	250.21 (12.21)	44.26 (15.10)	21/17	20/18

**Figure 2:**
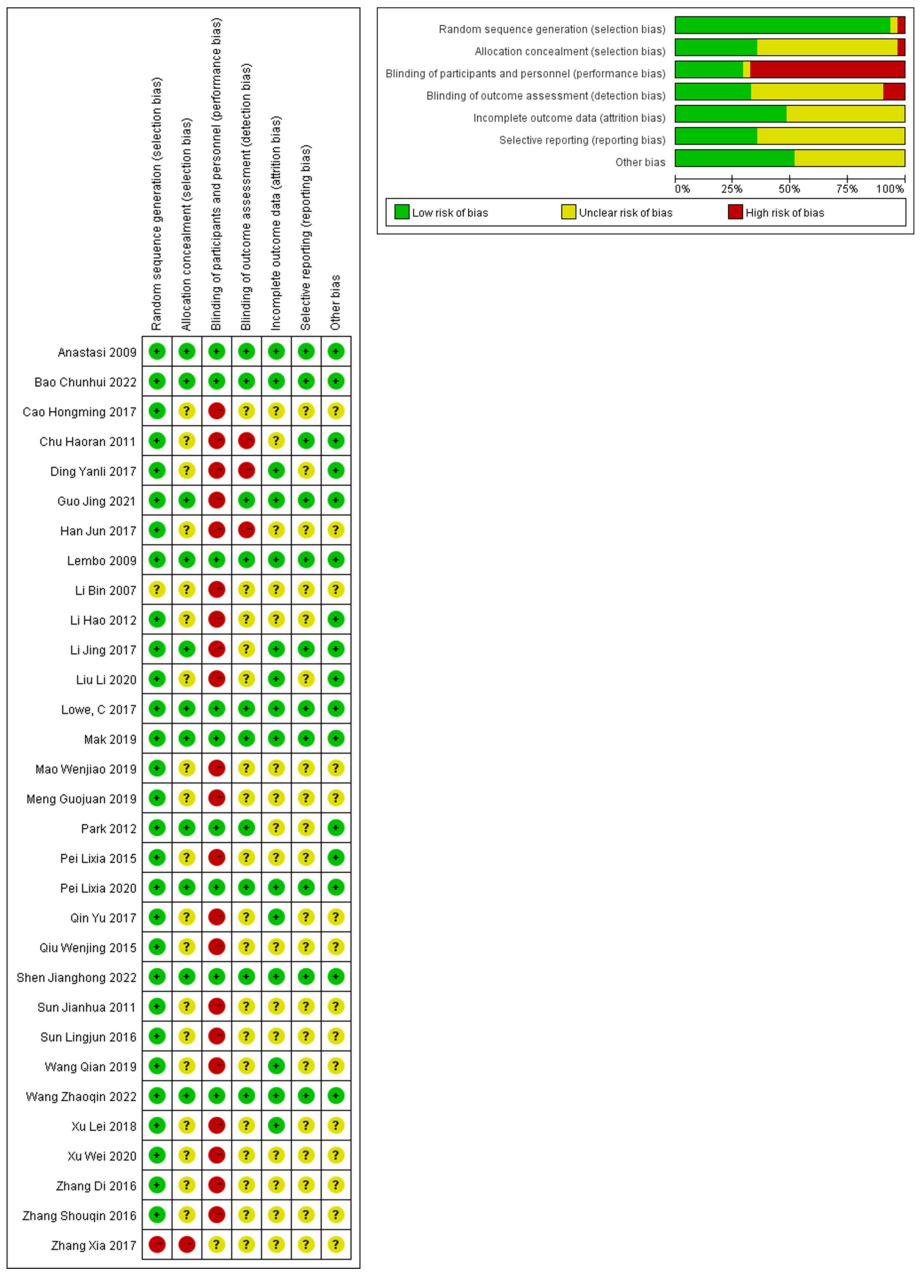
Summary of risk of bias.

Among the 31 trials included, 9 (21.43%) were sham controlled and 22 (78.57%) were open-label trials. Acupuncture was compared to pharmacological medications in fourteen of the 22 open-label trials, and in one trial, participants were divided into IBS-D and IBS-C groups, with pinaverium and polyethylene glycol serving as corresponding control interventions (12). Six of the 22 open-label trials compared moxibustion to medications, while two of the 22 trials compared the combination of acupuncture and moxibustion to pharmaceuticals. Seven sham controlled trials compared real acupuncture to sham acupuncture, Bifidobacterium tetralogy capsules were used with the same dosage between real and sham groups in one trial (38), another sham controlled trial compared real acupuncture plus real moxibustion to sham acupuncture and sham moxibustion (14), and two sham controlled trials compared moxibustion to sham moxibustion. Seven sham controlled studies were distinguished for their high quality, since each of the 7 domains of risk of bias was deemed to have a low risk. Detection bias existed in the residual trials due to participants not being blinded to the treatments and the primary and secondary outcomes being subjective. Therefore, it was thought that there was a high risk of bias in the 22 open-label trials (Figure 2).

### Primary outcome: Symptom severity

**Figure 3:**
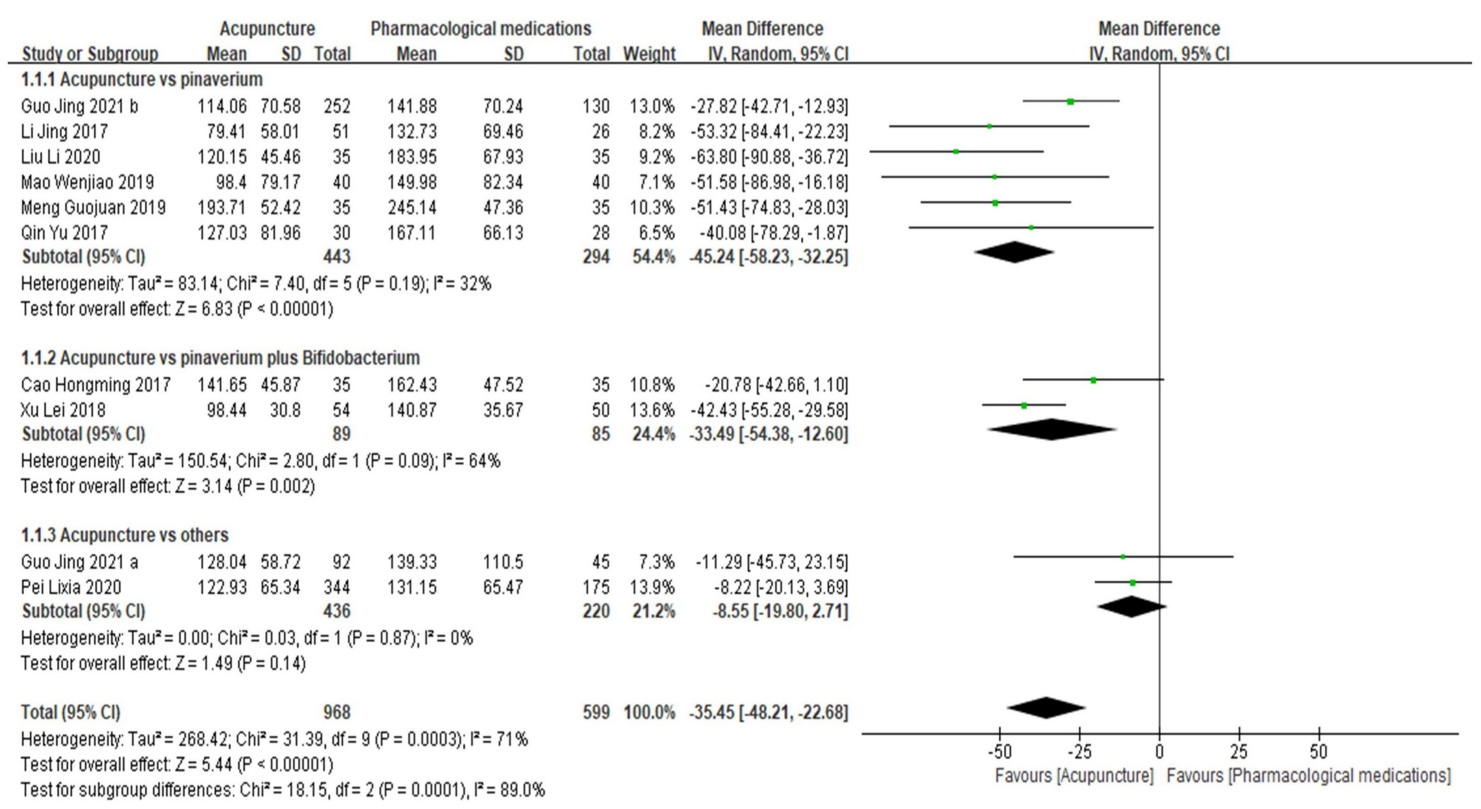
Forrest plot of acupuncture vs. pharmacological medications for symptom severity at the time of treatment ending. Results are shown by using the random-effect model with mean difference and 95% confidence intervals (CI).

**Figure 4:**
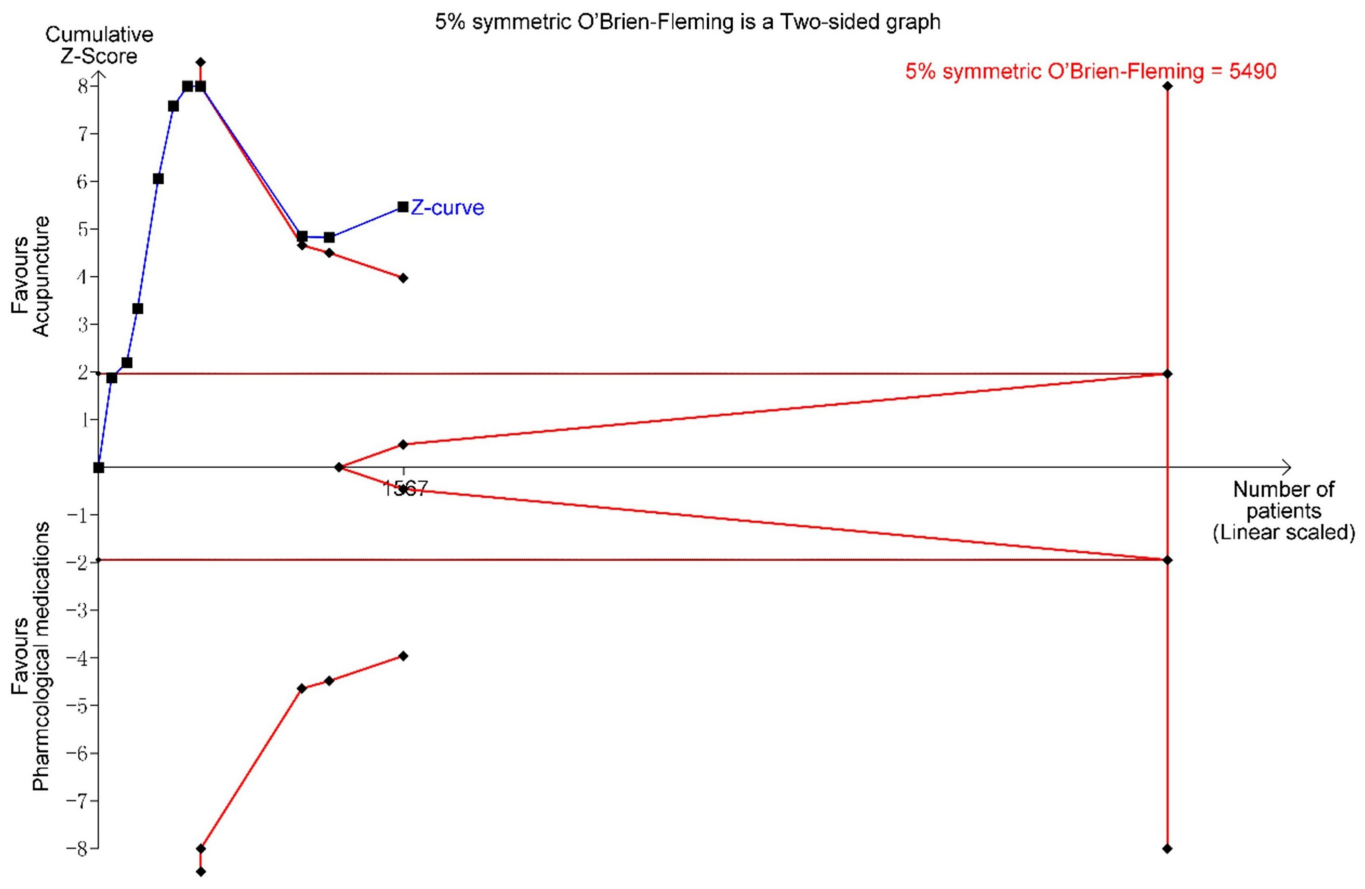
Trial sequential analysis (TSA) for symptom severity. Trial sequential analysis (TSA) of 10 trials comparing acupuncture with pharmacological medications for symptom severity in patients with IBS. The TSA shows that the information size is insufficient, but the cumulative Z score crossed O'Brien-Fleming alpha-spending significance boundaries. The evidence is sufficient to identify the effect of intervention. A required information size of 5,490 was calculated using α = 0.05 (two sided), ß = 0.20 (power 80%).

**Figure 5:**
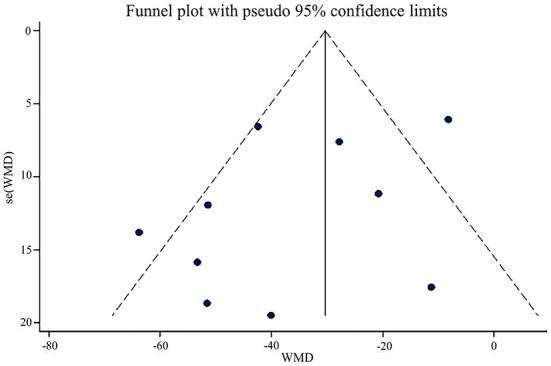
Funnel plot of acupuncture vs. pharmacological medications on IBS symptom severity at the time of treatment ending.

**Figure 6:**
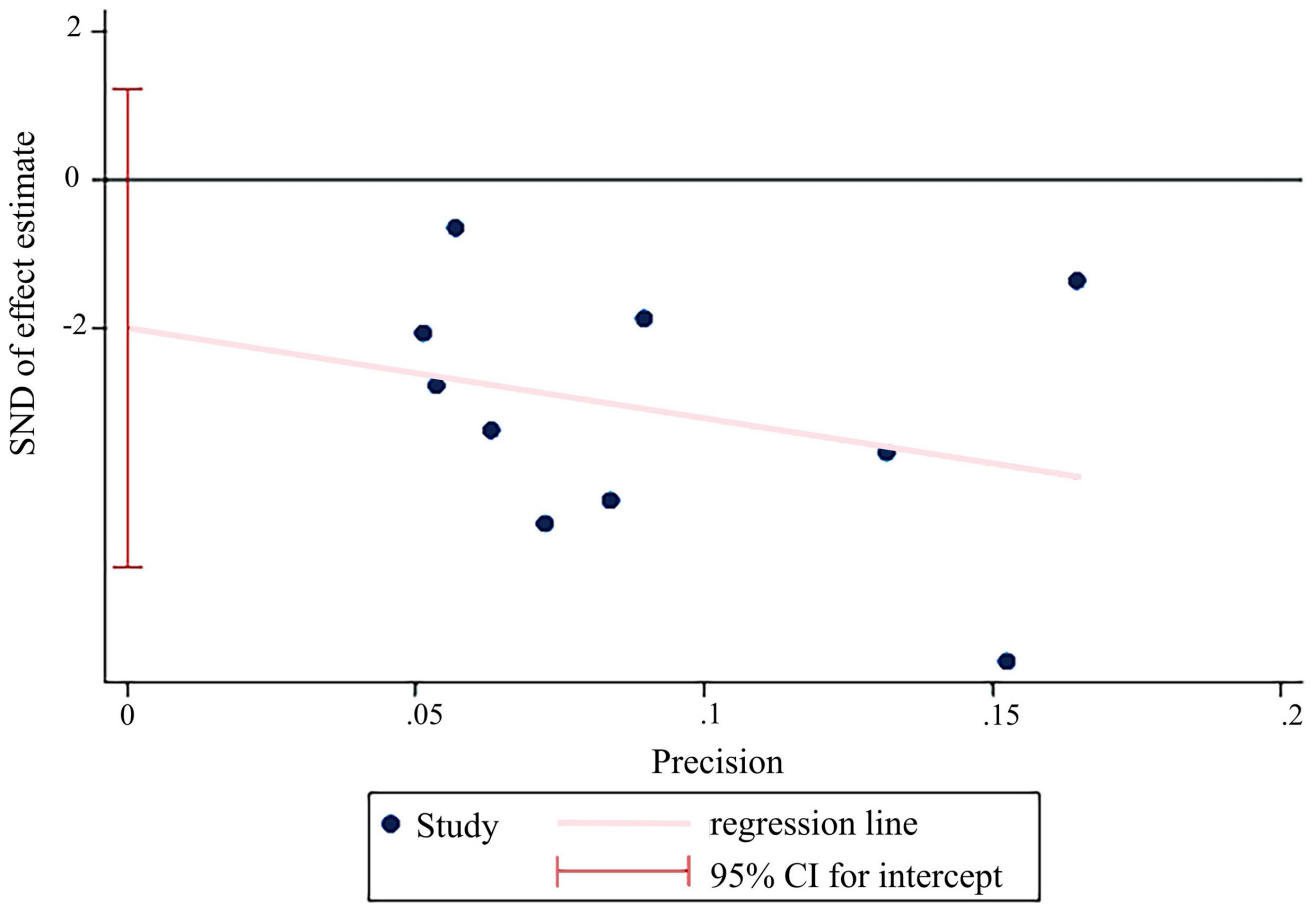
Egger test of acupuncture vs. pharmacological medications on IBS symptom severity at the time of treatment ending (Egger test, *P* = *0.191*).

**Figure 7:**
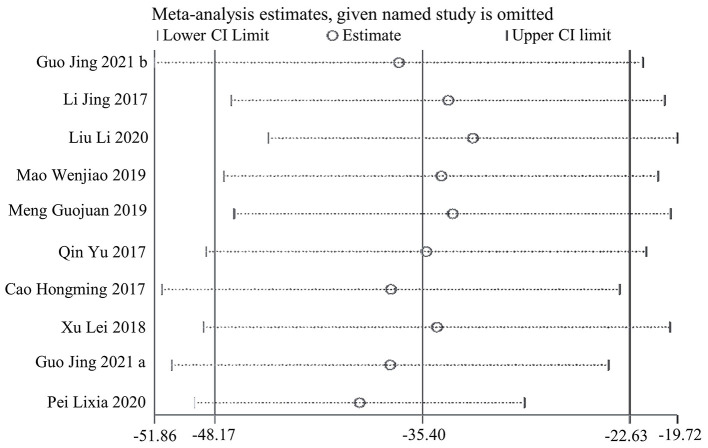
Sensitive analysis of acupuncture vs. pharmacological medications on IBS symptom severity at the time of treatment ending.

In terms of symptom severity at the time of treatment ending, pooled results from 9 articles containing 10 open-label trials showed a significant association between acupuncture and a reduction in symptom severity compared to pharmacological medications, along with substantial heterogeneity and moderate certainty (MD, −35.45; 95% CI, −48.21 to −22.68; *I*^2^ = 71%) (Figure 3 and Table 2). The exclusion of one outlier study (31) reduced heterogeneity with no significant change in results (MD, −39.30; 95% CI, −49.44 to −29.17; *I*^2^ = 39%). In trial sequential analysis (TSA), though the information size of participants with symptom severity didn't exceed the required information size (RIS), the cumulative Z score crossed O'Brien-Fleming alpha-spending significance boundaries (sample size, 1,567; RIS, 5,490) (Figure 4). Funnel plot analysis showed no asymmetry and suggested no publication bias (Figure 5). In addition, neither the Egger test (*P* = *0.191*) (Figure 6) nor the Begg test (*P* = *0.929*) (Supplementary Figure 1) detected any significant small study effects. Moreover, the result was robust in sensitivity analyses by systematically omitting one of the trials (Figure 7). At the time of follow-up, pooled results also indicated that acupuncture was superior to pharmacological medications on IBS symptom severity (MD, −23.8; 95% CI, −32.28 to −15.32, *I*^2^ = 24%) (Supplementary Figure 2). However, the therapeutic benefits of acupuncture on symptom severity are not exhibited when compared to sham acupuncture (SMD, 0.03, 95% CI, −0.25 to 0.31, *I*^2^ = 46%) (Supplementary Figure 3). With respect to moxibustion, results from one trial showed the association of symptom severity reduction and moxibustion rather than pharmacological medications with low certainty (MD, −59.75; 95% CI, −71.47 to −48.03) (Supplementary Figure 4). Results from two sham-controlled trials show a significant association between moxibustion and a reduction in symptom severity when compared to placebo moxibustion along with substantial heterogeneity and high certainty, not only at the time of treatment ending (SMD, −3.46, 95% CI, −5.66 to −1.27, *I*^2^ = 95%) but also at the time of follow-up (SMD, −4.07, 95% CI, −6.08 to −1.34, *I*^2^ = 96%) (Supplementary Figures 5, 6).

**Table 2:** Grade recommendation of acupuncture and moxibustion for IBS with different outcomes at the time of treatment ending.

**Certainty assessment**							**No. of patients**		**Effect**		**Certainty**	**importance**
**No. of** **studies**	**Study** **design**	**Risk of** **bias**	**Inconsistency**	**Indirectness**	**Imprecision**	**Other** **considerations**	**Acupuncture**	**Control** **therapy**	**Relative** **(95% CI)**	**Absolute** **(95% CI)**		
**Acupuncture vs. pharmacological medications for symptom severity at the time of treatment ending (assessed with: IBS-SSS)**												
10	Randomized trials	Very serious^a^	Not serious	Not serious	Not serious	Strong association	968	599	–	MD **35.45 fewer** (48.21 fewer to 22.68 fewer)	⊕⊕⊕○ Moderate	
**Moxibustion vs, pharmacological medications for symptom severity at the time of treatment ending (assessed with: IBS-SSS)**												
1	Randomized trials	Very serious^a^	Not serious	Not serious	Not serious	None	40	40	–	MD **59.75 lower** (71.47 lower to 48.03 lower)	⊕⊕○○ Low	
**Moxibustion vs. placebo moxibustion for symptom severity at the time of treatment ending (assessed with IBS-SSS)**												
2	Randomized trials	Not serious	Not serious	Not serious	Not serious	None	90	90	–	SMD **3.46 SD lower** (5.66 lower to 1.27 lower)	⊕⊕⊕⊕ High	
**Acupuncture vs. pharmacological medications for abdominal pain at the time of treatment ending**												
7	Randomized trials	Very serious^a^	Not serious	Not serious	Not serious	Strong association	562	366	–	SMD **0.35 SD lower** (0.48 lower to 0.21 lower)	⊕⊕⊕○ Moderate	
**Acupuncture vs. sham acupuncture for abdominal pain at time of treatment ending**												
4	Randomized trials	Serious^a^	Serious^b^	Not serious	Not serious	None	144	137	–	SMD **0.24 SD lower** (0.48 lower to 0.01 lower)	⊕⊕○○ Low	
**Moxibustion vs. pharmacological medications for abdominal pain at the time of treatment ending**												
3	Randomized trials	Very serious^a^	Not serious	Not serious	Not serious	None	96	96	–	SMD **0.75 SD lower** (1.04 lower to 0.46 lower)	⊕⊕○○ Low	
**Acupuncture and moxibustion vs. pharmacological medications for abdominal pain at the time of treatment ending**												
2	Randomized trials	Very serious^a^	Serious^c^	Not serious	Not serious	None	106	105	–	SMD **2 SD lower** (3.04 lower to 0.96 lower)	⊕○○○ Very low	
**Acupuncture and moxibustion vs. sham acupuncture and sham moxibustion for abdominal pain at the time of treatment ending**												
1	Randomized trials	Not serious	Not serious	Not serious	Very serious^d^	None	14	15	–	MD **1.12 lower** (1.78 lower to 0.46 lower)	⊕⊕○○ Low	
**Acupuncture vs. pharmacological medications for quality of life at the time of treatment ending (assessed with: IBS-QOL)**												
7	Randomized trials	Very serious^a^	Not serious	Not serious	Not serious	Strong association	838	497	–	MD **4.56 higher** (1.46 higher to 7.67 higher)	⊕⊕⊕○ Moderate	
**Moxibustion vs. pharmacological medications for quality of life at the time of treatment ending (assessed with: IBS-QOL)**												
3	Randomized trials	Very serious^a^	Not serious	Not serious	Not serious	None	119	118	–	MD **6.97 higher** (5.78 higher to 8.16 higher)	⊕⊕○○ Low	

CI, confidence interval; MD, mean difference; SMD, standardized mean difference;

a High risk of blinding and allocation concealment;

b Consequence was influenced when one of study was removed;

c Heterogeneity: I-squire 90%;

d Sample size is small and only one trial is available.

Subgroup analyses revealed that acupuncture was superior to pinaverium (MD, −45.24; 95% CI, −58.23 to −32.25; *I*^2^ = 32%) and pinaverium plus Bifidobacterium (MD, −33.49; 95% CI, −54.38 to −12.60; *I*^2^ = 64%) for symptom severity reduction (Figure 3). It also showed that there was no significant difference in symptom severity reduction between acupuncture and other medications like polyethylene glycol and lactulose oral liquid in patients with IBS-C (MD, −8.55; 94% CI, −19.80 to 2.71; *I*^2^ = 0%) (Figure 3).

### Secondary outcomes: Abdominal pain and quality of life

Data from 7 RCTs demonstrated that acupuncture is linked with a reduction in abdominal pain compared to pharmacological medications with heterogeneity and moderate certainty (SMD, −0.35; 95% CI, −0.48 to −0.21; *I*^2^ = 47%) (Supplementary Figure 7). Trial sequential analysis showed that the sample size of abdominal pain didn't transcend the required information size (RIS), but the cumulative Z score crossed O'Brien-Fleming alpha-spending significance boundaries (sample size, 928; RIS, 1,260) (Supplementary Figure 8).

Pooled results from four sham-controlled studies revealed a significant benefit of real acupuncture over sham acupuncture for the relief of abdominal pain, along with small heterogeneity and low certainty (SMD, −0.24; 95% CI, −0.48 to −0.01; *I*^2^ = 8%) (Supplementary Figure 9). Three open-label trials on moxibustion showed that the reduction of abdominal pain was associated with moxibustion in comparison with pharmacological medications without heterogeneity and low certainty (SMD, −0.75; 95% CI, −1.04 to −0.46; *I*^2^ = 0) (Supplementary Figure 10). Pooled results from two trials found that the reduction of abdominal pain was associated with a combination of acupuncture and moxibustion rather than pharmacological medications with considerable heterogeneity and very low certainty (SMD, −2.00; 95% CI, −3.04 to −0.96; *I*^2^ = 90%) (Supplementary Figure 11). A single sham-controlled trial revealed that acupuncture and moxibustion are superior to sham acupuncture and sham moxibustion with low certainty (MD, −1.12; 95% CI, −1.78 to −0.46) (Supplementary Figure 12). Two sham-controlled trials also show a significant association between moxibustion and a reduction in abdominal pain when compared to placebo moxibustion along with substantial heterogeneity at the time of treatment ending (SMD, −2.74, 95% CI, −4.81 to −0.67, *I*^2^ = 96%) and follow-up (SMD, −2.87, 95% CI, −4.75 to −0.99, *I*^2^ = 95%) (Supplementary Figures 13, 14).

Regarding quality of life (QOL) at the time of treatment ending, 6 papers containing 7 trials analyzing 1,335 participants contained data demonstrating the association between improvement of quality of life and acupuncture compared to pharmacological medications with substantial heterogeneity and moderate certainty (MD, 4.56; 95% CI, 1.46–7.67; *I*^2^ = 79%) (Supplementary Figure 15). The exclusion of two outliers (18, 24) reduced heterogeneity without reversing change in effect (MD, 2.37; 95% CI, 0.16–4.59; *I*^2^ = 43%). At the time of follow-up, pooled results also suggested that acupuncture is superior to pharmacological medications on improvement of quality of life (MD, 4.33; 95% CI, 2.54–6.11; *I*^2^ = 0%) (Supplementary Figure 16). Three open-label trials revealed that the improvement of QOL was associated with moxibustion compared with pharmacological medications with small heterogeneity and low certainty (MD, 6.97; 95% CI, 5.78–8.16, *I*^2^ = 21%) (Supplementary Figure 17).

## Discussion

In this meta-analysis of 31 randomized controlled trials, acupuncture or moxibustion were found to be beneficial for IBS symptom severity, abdominal pain and quality of life. The present study updated the synthesis of the current evidence and suggests that acupuncture or moxibustion could reduce symptom severity and abdominal pain, and improve quality of life with low to high certainty of evidence (Table 2).

### Principal findings and comparison with other studies

Although several systematic review and meta-analysis on acupuncture and moxibustion has been conducted (11, 44–47), primary outcome assessment of these studies has been limited to effectiveness rate. The continuous outcome from one of these reviews used Chinese Medicine symptoms integral with weighted mean difference (WMD) to estimate the efficacy (45). However, Chinese Medicine symptoms integral to IBS were rarely used in the clinical trials. The IBS series scales, such as the symptom severity scale (IBS-SSS) and quality of life (IBS-QOL), are widely used worldwide. Hence, symptom severity measured by IBS-SSS and quality of life measured by IBS-QOL are necessary to be assessed. Abdominal pain in the included trials was measured by Likert classification or visual analog scoring, the standardized scales should receive more application. Therefore, IBS series scales, such as IBS-SSS which including abdominal pain, are recommended in further research. Another essential issue which is not discussed in previous review is the increased type I error rate due to sparse data and repeated significance testing when updating meta-analysis with new trials.

Consistent with previous researches (11, 48), our results showed that acupuncture is linked with a significant reduction in symptom severity and abdominal pain in open-label studies. However, there may be an overestimation of treatment response in these trials due to a lack of blinding (48), since the therapeutic benefits of acupuncture on symptom severity are not exhibited when compared to sham acupuncture. Our analysis shows that real acupuncture is related to a reduction in abdominal pain compared to sham control, which differs from the findings of the previous review (11), owing to the inclusion of the latest published trials (38). However, the interesting finding was reversed when the sensitive analysis was conducted by omitting one of the trials (38), which indicated the result wasn't robust. What should be noted is that the sham control used in these trials, which involves skin penetrating needles inserted at non-acupuncture points, may have potential weak physiological activity that could influence the outcome and lead to a more feasible intervention response (11), particularly in the IBS population where high placebo responses are common (49). The ongoing randomized sham-controlled trials [NCT04276961 (50), ChiCTR2100044762] will clarify the efficacy of acupuncture compared with sham-acupuncture. And more shams, which are unlikely to have physiological effects, would be necessary for further research.

An exciting result, which is different from acupuncture's result, is found in moxibustion. On the one hand, our results show that moxibustion, like the pooled open-label trials result of acupuncture, is linked with a significant reduction in symptom severity and abdominal pain; on the other hand, the real and sham-controlled trials of moxibustion show benefits in symptom severity, which differ from the pooled sham-control trials result of acupuncture. Although the number of rigorous sham-controlled trial is limited and heterogeneity is great, ongoing RCTs (51) and the registered RCT with ID ChiCTR2100046852 may provide additional evidence of moxibustion's effectiveness for IBS treatment. Hence, more rigorous sham-controlled trials should be conducted in the future to enhance their reliability.

The major innovation that differentiated this research from prior reviews was the trial sequence analysis on IBS symptom severity and abdominal pain. Due to the potential for additional bias, heterogeneity in various features of the design and conduct of the included trials, and an inflated type I error rate, it is reasonable to interpret a meta-analysis with a higher level of skepticism than a single randomized controlled trial (52). Though the number of patients included in our study is much smaller than the calculated optimal information size, the cumulative Z score crossed O'Brien-Fleming alpha-spending significance boundaries for the outcomes of symptom severity and abdominal pain. Trial sequence analysis shows that there is enough evidence to show that acupuncture is better than pharmaceutical drugs when it comes to reducing symptoms and abdominal pain in open-label trials. More research is unlikely to change this result.

For a meta-analysis to provide definitive evidence, it must meet the basic requirements of a well-designed, adequately powered, and rigorously executed single randomized controlled trial (52). However, the majority of studies comparing acupuncture or moxibustion to pharmacological therapy are not blind. And expectation effects, which are defined as the impact of expectations on subjective outcomes, may differ between acupuncture and drug treatment (53, 54). As a result of the lack of blinding and differential expectations, it is impossible to determine whether any of the reported benefits of acupuncture are due to a larger biological effect of acupuncture needling compared to drugs, or the impact of the trial participants' greater expectation of benefit from acupuncture (11). Therefore, we recommend that future acupuncture and moxibustion studies should focus on sham-controlled trials. Further research should consider these questions as a research direction for some of the ideas we'd like to pursue but haven't been able to because of a lack of related trials, such as the effect difference of acupuncture and moxibustion between different subtypes of IBS and different regions.

### Strengths and limitations

This systematic review and meta-analysis possesses a number of methodological strengths. We followed the Cochrane Collaboration's recommendations and were registered in PROSPERO under the number CRD42021262118. This study also included a rigorous assessment of the quality of evidence using the GRADE approach. In addition, trial sequence analysis was used to evaluate the required information size and interim monitoring boundaries, which could decrease the probability of type I error.

Nonetheless, several limitations are unavoidable. First, the methodologic quality of the included trials was generally low due to a lack of blinding and allocation concealment, which limited the credibility of the results and contributed to a poorer evidence grade. Second, outcomes in our research were limited to symptom severity and quality of life, the possibility of a risk of selective bias should be considered. Thirdly, abdominal pain in the RCTs was measured by different classification scales, and the effect size was assessed with standard mean difference (SMD), which may have decreased the accuracy of the effect size. In light of these limitations, more sham-controlled trials of acupuncture or moxibustion are required to detect the treatment response and long-term prognosis.

## Conclusions

The findings of this systematic review and meta-analysis suggest that acupuncture and/or moxibustion are beneficial for symptom severity, abdominal pain and quality of life in IBS. The effects of acupuncture and moxibustion should be better known by more doctors and patients and widely used in clinical practice. However, in sham control trials, acupuncture hasn't exhibited robust and stable evidence, and moxibustion's results show great heterogeneity. Hence, more rigorous sham control trials of acupuncture or moxibustion are necessary.

## Data availability statement

The original contributions presented in the study are included in the article/Supplementary material, further inquiries can be directed to the corresponding author/s.

## Author contributions

SH and ZB conceived the study. HW and SQ designed the protocol. YY performed the literature search. KR and KZ selected the studies. YY and KR extracted the relevant information. YY and MS synthesized the data. YY wrote the first draft of the paper. All the authors critically revised successive drafts of the paper and approved the final version.

## Funding

This work is supported by the projects of the National Natural Science Foundation of China (Nos. 81974563 and 81904148), National Administration of Traditional Chinese Medicine of China (2019XZZX-XH002), Guangdong Provincial Hospital of Chinese Medicine for Specific Research (YN10101907), Basic and Applied Basic Research Projects of Guangzhou (202102010226, 202102010207), Collaborative Innovation Team Project of Guangzhou University of Chinese Medicine (2021xk63), Province Administration of Traditional Chinese Medicine of Guangdong (20192024, 20222074), Young innovative talents project of general colleges and universities in Guangdong Province (2019KQNCX023), and Guangdong Medical Science and Technology Research Fund Project (A2020181).

## Conflict of interest

Authors SH, HW, SQ, and HZ reported receiving grants the National Natural Science Foundation of China, internal funding from the Traditional Chinese Medicine Bureau of Guangdong Province during the conduct of the study and internal funding from the Guangdong Provincial Hospital of Chinese Medicine for Specific Research and Guangzhou University of Chinese Medicine. The remaining authors declare that the research was conducted in the absence of any commercial or financial relationships that could be construed as a potential conflict of interest.

## Publisher's note

All claims expressed in this article are solely those of the authors and do not necessarily represent those of their affiliated organizations, or those of the publisher, the editors and the reviewers. Any product that may be evaluated in this article, or claim that may be made by its manufacturer, is not guaranteed or endorsed by the publisher.
