# Supplementary material for: Clinical evidence of acupuncture and moxibustion for irritable bowel syndrome: A systematic review and meta-analysis of randomized controlled trials
Source: Front Public Health. 2022 Nov 24;10:1022145. doi: 10.3389/fpubh.2022.1022145 (PMC9801330; doi:10.3389/fpubh.2022.1022145)
Supplement: Supplementary file 2 [file Table_2.DOCX]

**Supplementary Figure 1**. Begg test of acupuncture vs pharmacological medications on IBS symptom severity at the time of treatment ending (Begg test, *P=0.929*)

**Supplementary Figure 2.** Forrest plot of acupuncture vs pharmacological medications on IBS symptom severity at the time of follow up; results are shown by using the fixed-effect model with mean difference and 95% confidence intervals (CI).


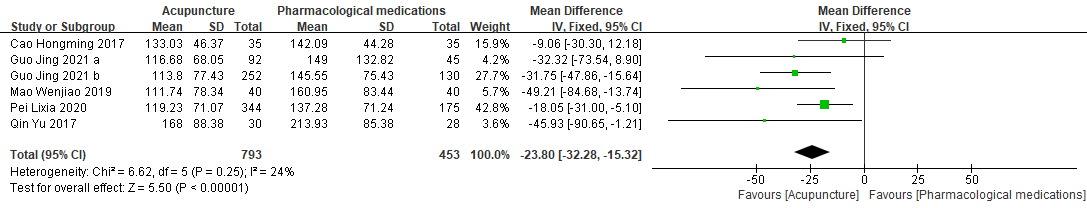


**Supplementary Figure 3.** Forrest plot of acupuncture vs sham acupuncture on IBS symptom severity scale at the time of treatment ending; results are shown by using the fixed-effect model with mean difference and 95% confidence intervals (CI).


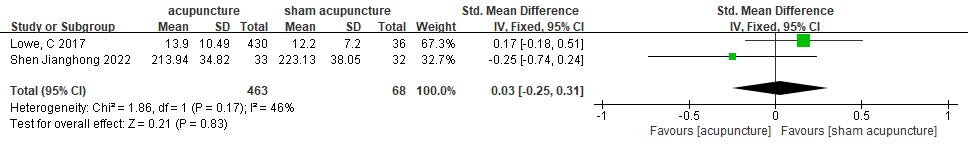


**Supplementary Figure 4.** Forrest plot of moxibustion vs pharmacological medications on IBS symptom severity at the time of treatment ending; results are shown by using the fixed-effect model with mean difference and 95% confidence intervals (CI).


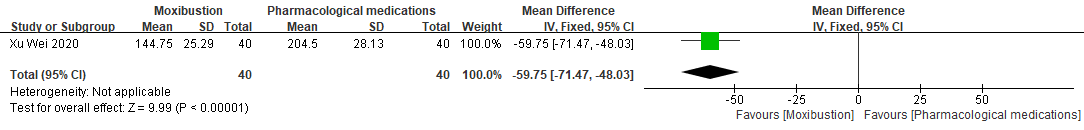


**Supplementary Figure 5.** Forrest plot of moxibustion vs sham moxibustion on IBS symptom severity scale at the time of treatment ending; results are shown by using the random-effect model with mean difference and 95% confidence intervals (CI).


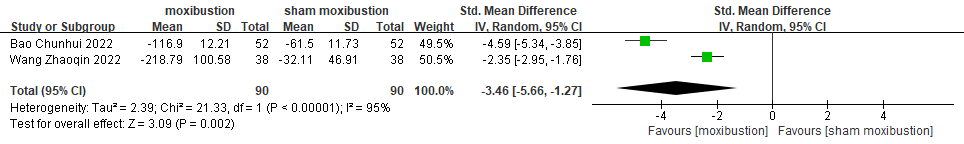


**Supplementary Figure 6.** Forrest plot of moxibustion vs sham moxibustion on IBS symptom severity scale at the time of follow up; results are shown by using the random-effect model with mean difference and 95% confidence intervals (CI).


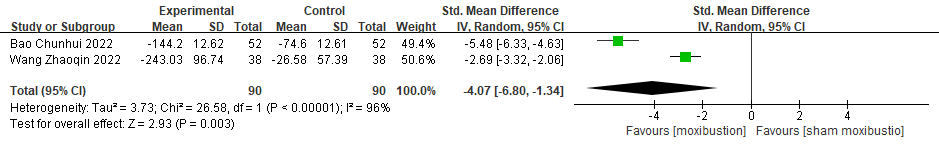


**Supplementary figure 7.** Forrest plot of acupuncture vs pharmacological medications on abdominal pain at the time of treatment ending; results are shown by using the fixed-effect model with standard mean difference and 95% confidence intervals (CI).


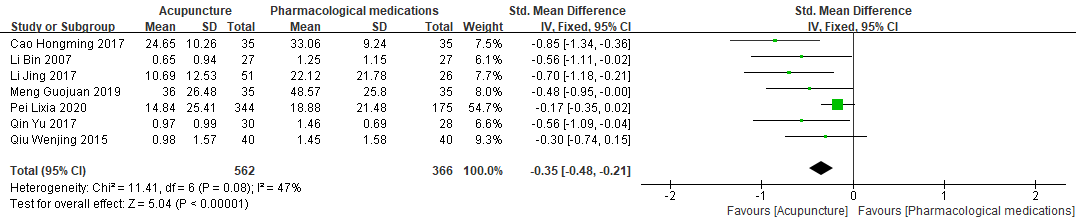


**Supplementary figure 8.** Trial Sequential Analysis (TSA) for Abdominal Pain

Trial sequential analysis (TSA) of 7 trials comparing acupuncture with pharmacological medications on abdominal pain. The TSA shows that the information size is insufficient, but the cumulative Z score crossed O’Brien-Fleming alpha-spending significance boundaries. The evidence is sufficient to identify the effect of intervention. A required information size of 1260 was calculated using ⍺ = 0.05 (two sided), ß = 0.20 (power 80%).


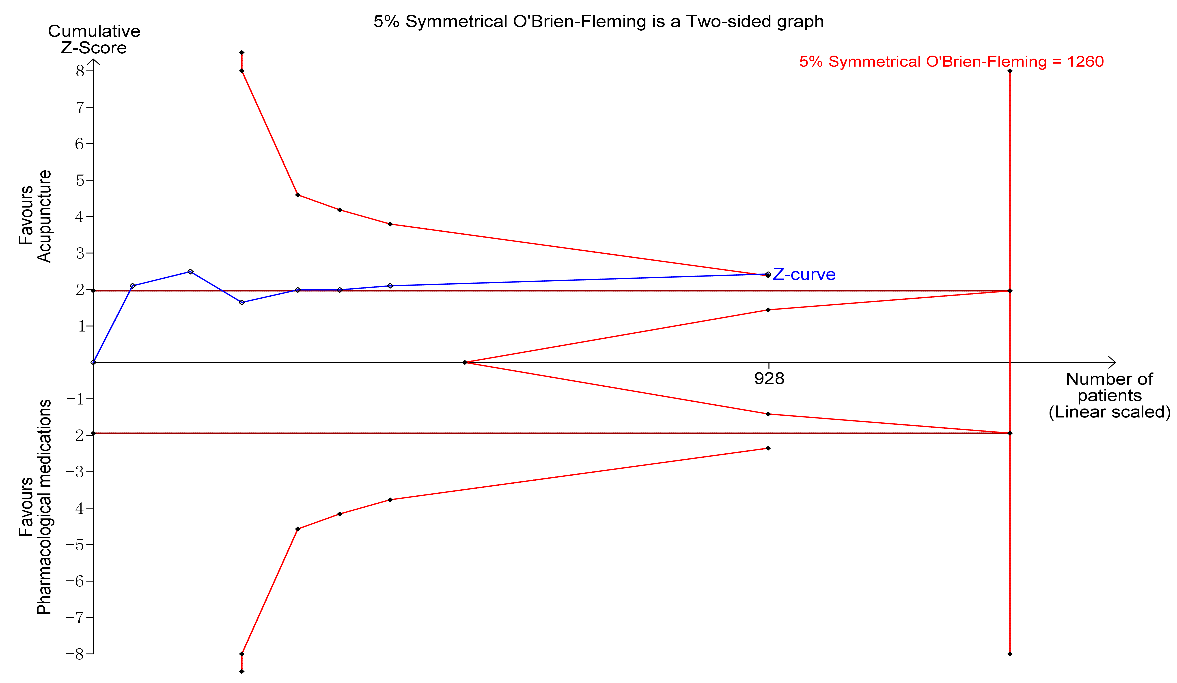


**Supplementary figure 9.** Forrest plot of acupuncture vs sham acupuncture on abdominal pain at the time of treatment ending; results are shown by using the fixed-effect model with standard mean difference and 95% confidence intervals (CI).


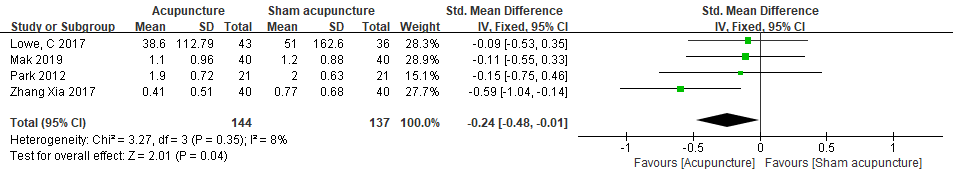


**Supplementary figure 10.** Forrest plot of moxibustion vs pharmacological medications on abdominal pain at the time of treatment ending; results are shown by using the fixed-effect model with standard mean difference and 95% confidence intervals (CI).


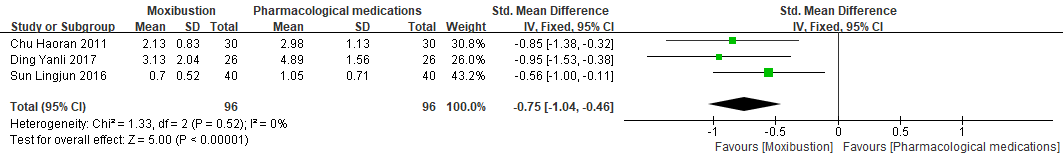


**Supplementary figure 11.** Forrest plot of the combination of acupuncture and moxibustion vs pharmacological medications on abdominal pain at the time of treatment ending; results are shown by using the random-effect model with standard mean difference and 95% confidence intervals (CI).


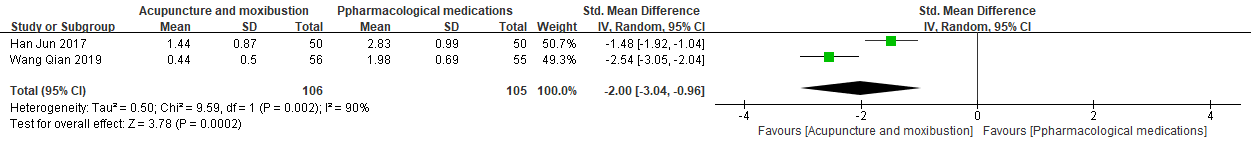


**Supplementary figure 12.** Forrest plot of acupuncture and moxibustion vs sham acupuncture and sham moxibustion on abdominal pain at the time of treatment ending; results are shown by using the fixed-effect model with mean difference and 95% confidence intervals (CI).


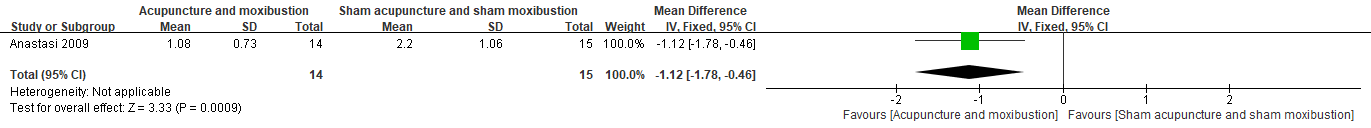


**Supplementary Figure 13.** Forrest plot of moxibustion vs sham moxibustion on abdominal pain at the time of treatment ending; results are shown by using the random-effect model with mean difference and 95% confidence intervals (CI).

**
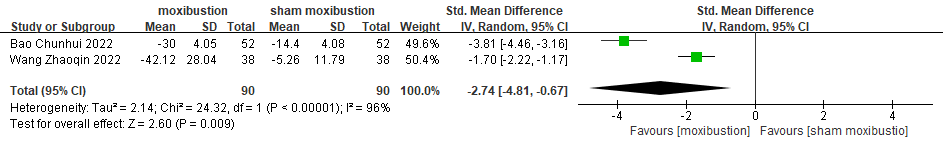
**

**Supplementary Figure 14.** Forrest plot of moxibustion vs sham moxibustion on abdominal pain at the time of follow up; results are shown by using the random-effect model with mean difference and 95% confidence intervals (CI).

**
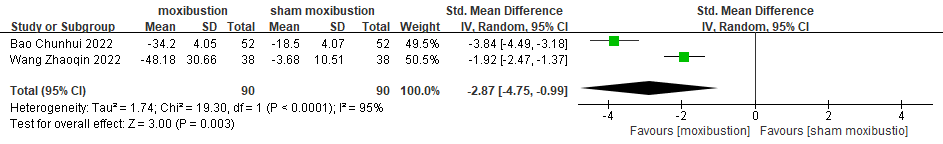
**

**Supplementary Figure 15.** Forrest plot of acupuncture vs pharmacological medications on quality of life at the time of treatment ending; results are shown by using the random-effect model with mean difference and 95% confidence intervals (CI).


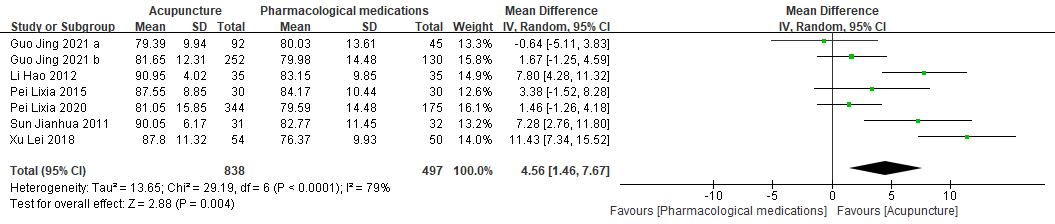


**Supplementary Figure 16.** Forrest plot of acupuncture vs pharmacological medications on quality of life at the time of follow up; results are shown by using the fixed-effect model with mean difference and 95% confidence intervals (CI).


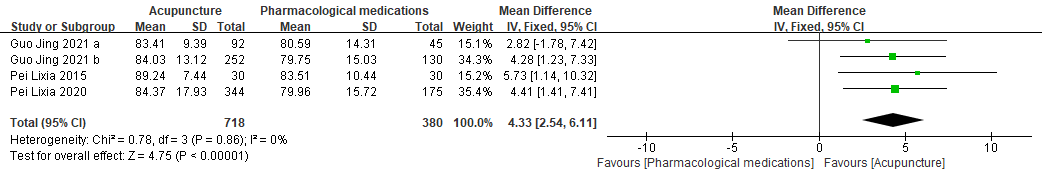


**Supplementary Figure 17.** Forrest plot of moxibustion vs pharmacological medications on quality of life at the time of treatment ending; results are shown by using the fixed-effect model with mean difference and 95% confidence intervals (CI).


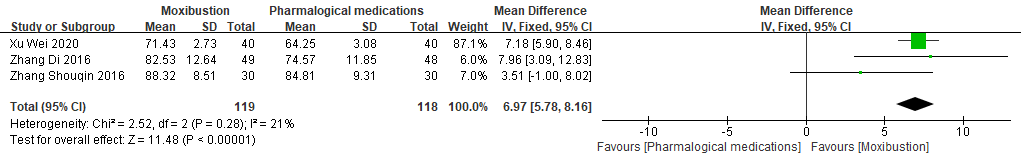


**Supplementary 18.** search strategy for PubMed

((((((((((("Irritable Bowel Syndrome"[Mesh]) OR (Irritable Bowel Syndrome[Title/Abstract])) OR (Syndrome, Irritable Bowel[Title/Abstract])) OR (Syndromes, Irritable Bowel[Title/Abstract])) OR (Colon, Irritable[Title/Abstract])) OR (Irritable Colon[Title/Abstract])) OR (Colitis, Mucous[Title/Abstract])) OR (Colitides, Mucous[Title/Abstract])) OR (Mucous Colitides[Title/Abstract])) OR (Mucous Colitis[Title/Abstract])) AND ((((((("Acupuncture"[Mesh]) OR (acupuncture*[Title/Abstract])) OR (Pharmacopuncture[Title/Abstract])) OR (electroacupuncture[Title/Abstract])) OR (electro-acupuncture[Title/Abstract])) OR (auriculoacupuncture[Title/Abstract])) OR (((((((("Moxibustion"[Mesh]) OR (moxibustion[Title/Abstract])) OR (moxa-moxibustion[Title/Abstract])) OR (bradycausis[Title/Abstract])) OR (byssocausis[Title/Abstract])) OR (acupuncture-moxibustion[Title/Abstract])) OR (electric moxibustion[Title/Abstract])) OR (electric-moxibustion[Title/Abstract])))) AND ((((((((((((((((("Random Allocation"[Mesh]) OR ("Clinical Trials as Topic"[Mesh])) OR ("Randomized Controlled Trials as Topic"[Mesh])) OR (random*[Title/Abstract])) OR (allocation, random[Title/Abstract])) OR (clinical trail[Title/Abstract])) OR (clinical trials, randomized[Title/abstract])) OR (trials, randomized clinical[Title/abstract])) OR (trial, randomized controlled[Title/abstract])) OR (controlled clinical trials, randomize[Title/abstract])) OR (controlled trial, randomized[Title/abstract])) OR (randomised controlled study[Title/abstract])) OR (randomised controlled trial[Title/abstract])) OR (randomized controlled study[Title/abstract])) OR (clinical trial[Publication Type])) OR (randomized controlled trial[Publication type])) OR (controlled clinical trial[Publication type]))
